# Combinatorial use of environmental stresses and genetic engineering to increase ethanol titres in cyanobacteria

**DOI:** 10.1186/s13068-021-02091-w

**Published:** 2021-12-17

**Authors:** Fraser Andrews, Matthew Faulkner, Helen S. Toogood, Nigel S. Scrutton

**Affiliations:** 1 EPSRC/BBSRC Future Biomanufacturing Research Hub, BBSRC/EPSRC Synthetic Biology Research Centre SYNBIOCHEM Manchester Institute of Biotechnology and School of Chemistry, The University of Manchester, Manchester, M1 7DN UK; 2 C3 Biotechnologies Ltd, 20 Mannin Way, Lancaster Business Park, Caton Road, Lancaster, LA1 3SW Lancashire UK

**Keywords:** Ethanol, Cyanobacteria, *Synechocystis* PCC 6803, Environmental stress, Carbon partitioning, Microbial pathway engineering, Synthetic biology

## Abstract

Current industrial bioethanol production by yeast through fermentation generates carbon dioxide. Carbon neutral bioethanol production by cyanobacteria uses biological fixation (photosynthesis) of carbon dioxide or other waste inorganic carbon sources, whilst being sustainable and renewable. The first ethanologenic cyanobacterial process was developed over two decades ago using *Synechococcus elongatus* PCC 7942, by incorporating the recombinant *pdc* and *adh* genes from *Zymomonas mobilis*. Further engineering has increased bioethanol titres 24-fold, yet current levels are far below what is required for industrial application. At the heart of the problem is that the rate of carbon fixation cannot be drastically accelerated and carbon partitioning towards bioethanol production impacts on cell fitness. Key progress has been achieved by increasing the precursor pyruvate levels intracellularly, upregulating synthetic genes and knocking out pathways competing for pyruvate. Studies have shown that cyanobacteria accumulate high proportions of carbon reserves that are mobilised under specific environmental stresses or through pathway engineering to increase ethanol production. When used in conjunction with specific genetic knockouts, they supply significantly more carbon for ethanol production. This review will discuss the progress in generating ethanologenic cyanobacteria through chassis engineering, and exploring the impact of environmental stresses on increasing carbon flux towards ethanol production.

## Background

The global reliance on the burning of fossil fuels for energy has led to increases in carbon dioxide and other greenhouse gas emissions, which is thought to be a primary driver of global climate change [1, 2]. Economically viable bio-based fuels and chemicals are essential for securing sustainable and renewable energy sources for future generations. In Europe, a major renewable transportation fuel is bioethanol, second only to biodiesel [3, 4]. The high-octane properties of bioethanol mean it is primarily used as a drop-in fuel for gasoline [5], with the bioethanol fraction varying from 10% (E10) to 51–83% (E85 fuels) [6]. The major bioethanol producers are the United States, Brazil and the European Union (29 billion gallons in 2021 [6]), which rely on the fermentation of first-generation biomass feedstocks of corn or sugar cane by engineered yeast [7].

In the circular economy model, there must be a balance between waste generation and recycling, to minimise any net impact on global challenges, such as climate change and other polluting practices. However, the production of bioethanol and its subsequent combustion both generate carbon dioxide, and it is unclear whether this is fully offset by carbon dioxide capture during biomass cultivation to yield a net carbon neutral footprint [8]. An alternative solution is to make bioethanol directly from biological carbon dioxide fixation (photosynthesis), rather than via secondary CO_2_-evolving fermentation on plant biomass. This would eliminate the land burden for biomass cultivation and the costs of subsequent processing to release hydrolysable sugars for fermentation [9, 10].

**Fig. 1:**
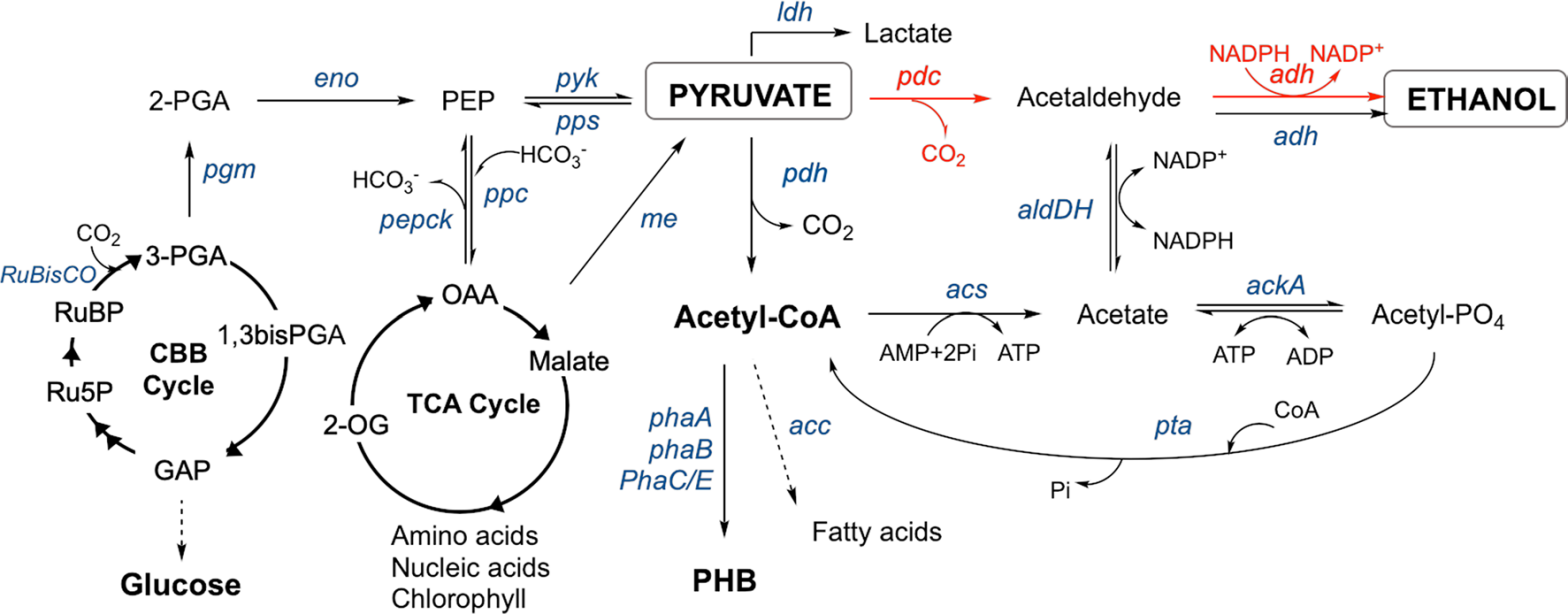
Native and engineered routes from carbon dioxide to ethanol in *Synechocystis*. Additional pathways are shown that provide flux through pyruvate. The pathway in red is the engineered ethanologenic route from *Zymomonas mobilis*. Pathway intermediates: GAP, glyceraldehyde-3-phosphate; OAA, oxaloacetate; 2-OG, α-ketoglutarate; PEP, phosphoenolpyruvate; 2-PGA, 2-phosphoglycerate; 3-PGA, 3-phosphoglycerate; PHB, polyhydroxybutyrate; RuBP, ribulose bisphosphate. Enzymes/genes: *acc*, acetyl-CoA carboxylase; *acs*, acetyl-CoA-synthase; *ackA*, acetate kinase; *adh*, alcohol dehydrogenase; *aldDH*, aldehyde dehydrogenase; *eno*, enolase; *ldh*, lactate dehydrogenase; me, malic enzyme; *Pdc*: pyruvate decarboxylase; *pdh*, pyruvate dehydrogenase complex; *pepck*, phosphoenolpyruvate carboxykinase; *pgm*, 2,3-bisphosphoglycerate-independent phosphoglycerate mutase; *phaA*, acetyl-CoA acetyltransferase; *phaB*, acetoacetyl-CoA reductase; *PhaC/E*, poly(3-hydroxyalkanoate) polymerase; *ppc*, phosphoenolpyruvate carboxylase; *pps*, phosphoenolpyruvate synthase; *pta*, phosphate acetyltransferase; *pyk*, pyruvate kinase; *RuBisCO*, ribulose-1,5-biphosphate carboxylase/oxygenase. Lactate and ethanol are both readily secreted by *Synechocystis.j*

Cyanobacteria (blue-green algae), such as *Synechococcus* [11] and *Synechocystis* [12], are potentially suitable chassis for photosynthetic ethanol production. These model microorganisms have a fully characterised genome, have established molecular biology techniques and are amenable to genetic manipulation [13]. Some cyanobacteria contain a genomic encoded endogenous ethanologenic pathway [14] (Fig. 1); however, ethanol production has only been detected after the incorporation of recombinant ethanologenic genes [15, 16]. These microorganisms have a higher photosynthetic rate than plants and algae (10% solar energy uptake into biomass), leading to a greater yield potential per-acre compared to traditional food crops [17]. They have the added advantage of being able to grow in brackish/industrial wastewater [18, 19], seawater [18] or brine [20].

In general, autotrophic (carbon fixation) microbial chassis generate lower titres of secondary products compared to heterotrophs. However, the superior carbon fixation credentials of cyanobacteria make exploratory investigations into their potential as bioethanol chassis worth pursuing. For example, a microbial factory could be established to generate bioethanol from the direct capture of carbon dioxide from the waste flues of heavy industry [21]. Secondary cyanobacterial products with significant economic value [17, 22] could be harvested (e.g. biofertiliser from biomass or biooil into biodiesel [23]), which could push the process into commercial viability. Given the large disparity between autotrophic and heterotrophic bioethanol titres, cyanobacterial chassis optimisation must go beyond the simple addition of an ethanologenic cassette to approach *S. cerevisiae* productivity. This review will discuss the current progress in engineering the cyanobacteria *Synechocystis* and *Synechococcus* for bioethanol and to a lesser extent other biofuels. It will include the optimisation of a heterologous ethanologenic cassette, and chassis redesign to maximise the supply of pyruvate, a major precursor for ethanol formation. In addition, the impact of environmental stresses on flux through pyruvate will be discussed, as will the importance of optimising the carbon partitioning between biomass and ethanol production.

### Engineering an ethanologenic cassette

The classical ethanologenic cassette for microbial heterologous expression is composed of pyruvate decarboxylase (pdc) and alcohol dehydrogenase II (adh) from microorganisms, such as *Zymomonas mobilis* (Fig. 1, highlighted in red; [15]). This pathway requires the cell to supply adequate quantities of the central metabolite pyruvate, which is generated from the Calvin–Benson–Bassham (CBB) cycle intermediate 3-phosphoglycerate via three enzymatic steps [15]. In this recombinant pathway, pyruvate undergoes decarboxylation by pdc to form acetaldehyde, with is subsequently reduced to ethanol via an NADPH-dependent adh (Fig. 1). Cyanobacteria do not contain a native pdc; however, there is a putative route to ethanol via acetaldehyde formation, which requires decarboxylation to acetyl-CoA via pyruvate oxidoreductase (pyrOR), followed by acetyl-CoA-synthase (acs)-dependent acetate formation [24]. Acetate could potentially be oxidised to acetaldehyde by a reversible aldehyde dehydrogenase (aldDH), and the native adh could generate ethanol as the final product (Fig. 1) [24].

In the first reported example, recombinant *pdc* and *adh* genes from *Z. mobilis* were highly expressed in *Synechococcus elongatus* PCC 7942 (*Syn*-7942) under the control of the cyanobacterial ribulose-1,5-bisphosphate carboxylase/oxygenase (RuBisCO) promoter [15]. However, ethanol production rates of only 6 mmol/OD_730nm_/L/day were achieved (~ 0.23 g/L in 28 days; Table 1) [15], far below the best performing engineered *Escherichia coli* (46 g/L in 48 h; [25]) and *S. cerevisiae* constructs (130 g/L ethanol in 65 h with *Saccharomyces cerevisiae* [26]). This equated to over a 100-fold reduction in the theoretical maximum titre that could be achieved based on the known expression levels of the two recombinant enzymes. This suggests that a pyruvate supply limitation may be present, and shows the potential of optimisation studies to dramatically increase bioethanol titres.

**Table 1:** Ethanol production by engineered cyanobacteria

Cyanobacterium	Ethanologenic cassette	Genotype	Ethanol rate (g/L/d)	Comments	Refs.
*Synechocystis* strain					
PCC 6803	pdc_Zm_ + adh_Syn_ (slr1192)	*glgC* + *phaC* + *phaE*	0.986	Dual copies; 2.96 g/L in 3 d; highest OD_730_ ~ 50; *pnblA* promoter	[27]
			~ 0.2	Dual copies; 5.5 g/L in 26 d; highest OD_730_ ~ 4; *pnblA* promoter; Nitrogen starvation	[27]
		*phaA* + *phaB*	0.285	Dual copies; 2.6 g/L in 9 d; *ppsbAII* promoter	[28, 29]
			0.255	Optimised pdc:adh expression ratio to 2:1; 2.3 g/L in 9 d; *prbc* promoter	[30]
			0.212	Dual copies; 5.5 g/L in 26 d; highest OD_730_ ~ 12; *prbc* promoter	[24]
		Plasmid	0.06	Overexpressed FBA and TK; 1.2 g/L in 20 d; *pnrsB* promoter for pdc and adh	[31]
			0.236	Patented; 7.1 g/L in 30 d; *ziaA* promoter; construction #1318	[32]
			0.235	Patented; 4.7 g/L in 20 d; *corT* promoter	[32]
			0.261	4.7 g/L in 18 d; highest OD_730_ ~ 13; pVZ325 plasmid; *petJ* promoter	[33]
			0.097	Patented; 3.6 g/L in 37 d; pVZ321b plasmid and others; *petJ* promoter	[34]
	pdc_Zm_ + adhII_Zm_	*pdc/adhII*	0.0766	0.46 g/L in 6 d; *psbA2* promoter	[35]
	pdc_Sc_ + adh_Syn_ (*slr0942*)	*glgC* + *phaA*	0.157	Co-culture of knockout & ethanologenic strains; 4.7 g/L in 30 d; *psbA* promoter	[36]
			0.137	4.1 g/L in 30 d; *psbA* promoter	[36]
*Synechococcus strain*					
PCC 7002	pdc_Zm_ + adh_Syn_ (slr1192)	*glgA1* + *glgA2*	0.220	Dual copies; 2.2 g/L in 10 d; highest OD_730_ ~ 4; *prbc* promoter	[37]
	-	-	0.410	Patented, JCC1581_B isolate; 5.62 g/L in 13.7 d;	[38]
PCC 7942^1^	pdc_Zm_ + adh_Zm_	Genomic	0.076	25% CO_2_ sparging; 0.23 g/L in 3 d; *prbcL* promoter	[39]
		Plasmid	0.008	0.23 g/L in 28 d; *prbsLS* promoter	[15]
*ABICyano1*^*2*^	-	Plasmid	0.552	Patented, plasmid TK504; copper inducible promoter	[40]

^1^*Synechococcus elongatus* strain; ^2^Phylogeny of the novel cyanobacterial isolate ABICyano1 is closest to *Cyanobacterium aponinum* PCC 10,605 and *Cyanobacterium aponinum* ETS-03 [16]

After proof of principle demonstration of a functional ethanologenic cassette in *Synechococcus*, the next stage is to assess *pdc* and *adh* homologues to observe if significant titre improvements can be achieved. An early win was the demonstration of a 50% improvement in ethanol titres by the substitution of the adh_*Zm*_ gene from *Z. mobilis* for the endogenous gene (slr1192; adh_*Syn*_) of *Synechocystis* sp. PCC 6803 (*Syn*-6803) [24]. This cassette was integrated within two sites on the chromosome, creating the *Syn*-HZ24 strain which produced ethanol titres of 5.5 g/L, equating to a productivity of ~ 0.2 g/L/d (Table 1). Similarly, the engineered UL030 strain contained two copies of the pdc_*Zm* +_ adh_*Syn*_ cassette integrated at a different neutral site within the genome. This displayed the highest published ethanol productivity of 0.29 g/L/d [29]. This approach of multiple ethanologenic cassette insertions within the genome of *Synechocystis* has become a proven strategy to increase ethanol titres [24, 28, 29, 37, 41]. However, plasmid-based ethanol-producing systems are also quite successful in cyanobacteria, and have often led to relatively high ethanol titres [33, 40, 42]. For example, the patented cyanobacterial strain, ABICyanol1, has achieved an ethanol productivity of 0.55 g/L/d using a plasmid-based system [40].

One possible explanation for the increased efficiency of adh_Syn_ over adh_Zm_ is that the former can utilise both NADH and NADPH as cofactors, whilst having a 74,000-fold greater activity with NADPH [24]. Given that *Syn*-6803 has ten-fold higher NADPH concentrations than NADH [43], this suggests adh_*Syn*_ is better suited for ethanol production in this host. Flux control through the CBB pathway was most highly dependent on the energy supply (ATP), and to a lesser extent by cofactor supply (NADPH) [77]. An *in silico* study generated a *Synechocystis* metabolic model designed for maximal ethanol production by increasing the coenzyme (NADPH) supply for adh, rather than redirecting pyruvate flux away from competing pathways [44]. The theoretical M2 strain was designed containing thirteen genetic modifications, most of which were deletions of enzymes that compete for NADPH. The theoretical ethanol yield for this chassis was 1.165 g ethanol/DCW/day, which is 57% of the theoretical maximal yield [44]. However, this extreme deletion strain has not been tested in *Synechocystis*, so it is unknown what impact the deletion of so many NADPH-dependent enzymes will have on its overall metabolism and biomass production, with likely knock-on effects for ethanol production.

Other adh enzymes trialled included the highly active NADPH-dependent aldehyde reductase (yqhD) from *E. coli* [45]. However, a comparison found adh_*Syn*_ to be more effective than yqhD at reducing isobutyraldehyde to isobutanol in an isobutanol-producing *Syn*-6803 strain [46]. Since this reaction is analogous to the reduction of acetaldehyde to ethanol, adh_*Syn*_ likely remains the most suitable enzyme for a *Synechocystis* ethanologenic cassette, although the engineering of improved adh and pdc enzymes may also be required to reach titres suitable for efficient recovery [47].

In vitro titration assays of the pdc_Zm_–adh_*Syn*_ pathway showed that pdc_*Zm*_ displays a relatively higher K_m_ and V_max_ than adh_*Syn*_, suggesting that acetaldehyde formation by pdc is the main rate-limiting step [30]. The catalytic efficiency of *Synechocystis* adh_*Syn*_ is higher than that of pdc_*Zm*_ because of a substantially lower *K*_d_, yet similar *V*_max_ values. Another study showed that although the abundance of both pdc and adh appear to influence ethanol titres, pdc abundance had a much stronger correlation with ethanol production, with R^2^ values of 0.89 and 0.44, respectively [48]. Whilst the pdc_*Zm*_ from *Z. mobilis* is currently the most efficient homologue known [49], other homologues have been trialled within the ethanologenic cassette. One study incorporated the pdc from *S. cerevisiae* into *Syn*-6803, as this enzyme shows improved functionality under acidic conditions induced by CO_2_ enriched sparging [36]. Unfortunately, no functional comparison between the two pdc enzymes was performed so the better homologue for cyanobacteria is unknown. The pdc from *Zymobacter palmae* boasts a lower *K*_m_ value for pyruvate than pdc_*Zm*_; however, a functional comparison between the two showed that pdc_*Zm*_ displays higher ethanol titres in *Syn*-6803 [50]. Instead of changing pdc homologue, enhancing pdc overexpression by optimising the promoters or ribosome binding sites within the construct may be a better strategy [42, 48, 50–52].

Fine tuning of ethanol production can be performed by optimising the balance of pdc_*Zm*_:adh_*Syn*_ expression levels. This is in line with the observation that the pyruvate to acetaldehyde step is rate limiting, so increasing the pdc_Zm_:adh_*Syn*_ concentration ratio could be a target for pathway optimisation [30]. Within the pdc_Zm_–adh_*Syn*_ system, titration assay studies found that the most economical and balanced expression ratio of the two enzymes is likely to be around 1:1.5 to 1:2, compared to the existing ratios of 1:30 to 1:40 (Table 1) [30]. An attempt was made to improve the ethanol titres by comparing three *Synechocystis* strains containing genomic integrated copies of pdc and adh at ratios of 1:1 (*Syn*-ZG25 and *Syn*-HZ24; 1 and 2 copies of both genes, respectively) and 2:1 (*Syn*-YQ4 with 2 copies of pdc_Zm_). Unfortunately, this study showed no significant difference in ethanol titres between dual site integrated strains with a ratio of 1:1 and 2:1 (2.2–2.3 g/L). This is perhaps not surprising given the expression levels showed very high levels of adh_*Syn*_ expression compared to pdc_Zm_, even in the 2:1 strain. The most significant finding was observed by comparing the pdc_Zm_–adh_*Syn*_ cassette copy number, with the single 1:1 integrated strain (*Syn*-ZG25) showing only 1.2 g/L ethanol titres after 9 days compared to 2.2–2.3 g/L for the double integrated *Syn*-YQ4 and *Syn*-HZ24 strains [30].

## Modulating flux through pyruvate

### The carbon partitioning problem

A fundamental problem which limits microbial biofuels production is the partitioning of fixed carbon between cell growth and metabolic maintenance and secondary product accumulation. This has been described in a recent review by Liu et al. [53] in a section discussing the optimisation of butanol production by editing native carbon flux [53]. This is compounded by microbial carbon fixation being inherently slow and inefficient compared to heterotrophic fixed carbon uptake and catabolism [54]. As downstream carbon consuming reactions are even slower, this limits the ability to increase carbon fixation efficiency. One approach to increasing carbon flux is to introduce a ‘carbon sink’, which is a high flux pathway that converts fixed carbon into a secondary molecule (e.g. bioethanol) that is secreted from the cell (Fig. 1). This effectively decouples the CBB cycle rate from growth and cell maintenance, and could potentially lead to increased efficiency of carbon dioxide fixation and light capture steps [54, 55].

Heterotrophic conversion of glucose to ethanol requires eight enzymatic steps; at least twenty-seven reactions are needed for ethanol production from CO_2_ in photosynthetic microorganisms [54]. As a result, unbalanced carbon partitioning in favour of bioethanol or other biofuel overproduction can create a carbon drained state where CBB cycle metabolites are depleted, leading to a decreased productivity and growth rate [56, 57]. As expected, studies have shown that increasing the carbon partitioning towards bioethanol production results in a proportional decrease in cellular biomass production [12, 30]. Counterintuitively, introduction of a carbon sink can actually increase total carbon productivity, which is defined as the total amount of carbon in the cellular biomass combined with the secreted carbon sink metabolites [54, 55]. Addition of a carbon sink appears to change various facets of cyanobacterial physiology, which may include increasing photosynthetic efficiency, carbon fixation capacity and chlorophyll content, all of which may contribute towards increased total carbon productivity [36, 55, 57]. Unfortunately, this effect appears to be somewhat of a double-edged sword since carbon partitioning values that are too high can reduce growth rates and lower total product evolution [33, 54, 57, 58].

A 2,3-butanediol (2,3-BD)-producing *Syn*-7942 strain was investigated to ascertain the likely optimal carbon partitioning ratio for maximal biofuel production [54]. A library of variant strains with different carbon partition ratios were screened for maximal 2,3-BD titres. The productivity maxima was found when 30% of carbon was partitioned towards 2,3-BD [54]. This limitation was also observed during the cyanobacterial production of acetyl-CoA-derived solar fuels and chemicals [59]. Since 2,3-BD and ethanol are both derived from pyruvate via a synthetic pathway involving decarboxylation and reduction, it is likely that this 30% value could be used as a yardstick for future development of ethanologenic strains. This could be particularly useful for the application of ethanol production in a continuous culture strategy. For example, during log growth phase of *Syn*-6803 the carbon fixation rate (2.1 g CO_2_/L/d) could lead to a theoretical maximal 0.22 g/L/d ethanol production when imposing a 30% carbon partitioning rate [60]. Some published ethanol productivities have already exceeded this theoretical maximum (Table 1), likely due to increased cell culture density prior to ethanol production initiation. Secondary product formation is likely to be higher during stationary phase, when carbon partitioning towards cellular growth is minimal.

The effect of excessive carbon partitioning on intracellular carbon limitation was observed during a study of a *Synechococcus sp.* PCC 7002 (*Syn*-7002) strain under diurnal light conditions [57]. Under batch cultivation conditions, both ethanol production and growth rates declined compared to wild-type cells due to an imbalance in the partitioning between carbon fixation and carbon sink production [57]. This demonstrates that when the carbon partitioning ratio towards ethanol was too high, key metabolite pools, such as 3-phosphoglycerate, 2-phosphoglycerate and phosphoenolpyruvate, likely became depleted to compensate for the lack of carbon supply. This carbon depletion eventually extended further into the core metabolism during prolonged batch cultivation, reducing the pool sizes of the citric acid (TCA) and CBB cycle metabolites, amino acids, as well as causing large-scale reduction in enzyme abundance [57]. However, whilst the photosynthetic capacity decreased, carbon partitioning towards ethanol increased from ~ 50% to ~ 80% after 10 days. This correlated with an increasing pdc activity throughout the 30-day experiment [57]. Increased partitioning towards ethanol likely contributed to an acceleration towards a non-viable, fixed carbon depleted state [57], although culture carbon dioxide levels were maintained in excess. Therefore, maintenance of an optimal carbon partitioning ratio is likely a key factor to optimising ethanol titres during prolonged cultivations.

### Carbon fixation modulation towards pyruvate accumulation

**Fig. 2:**
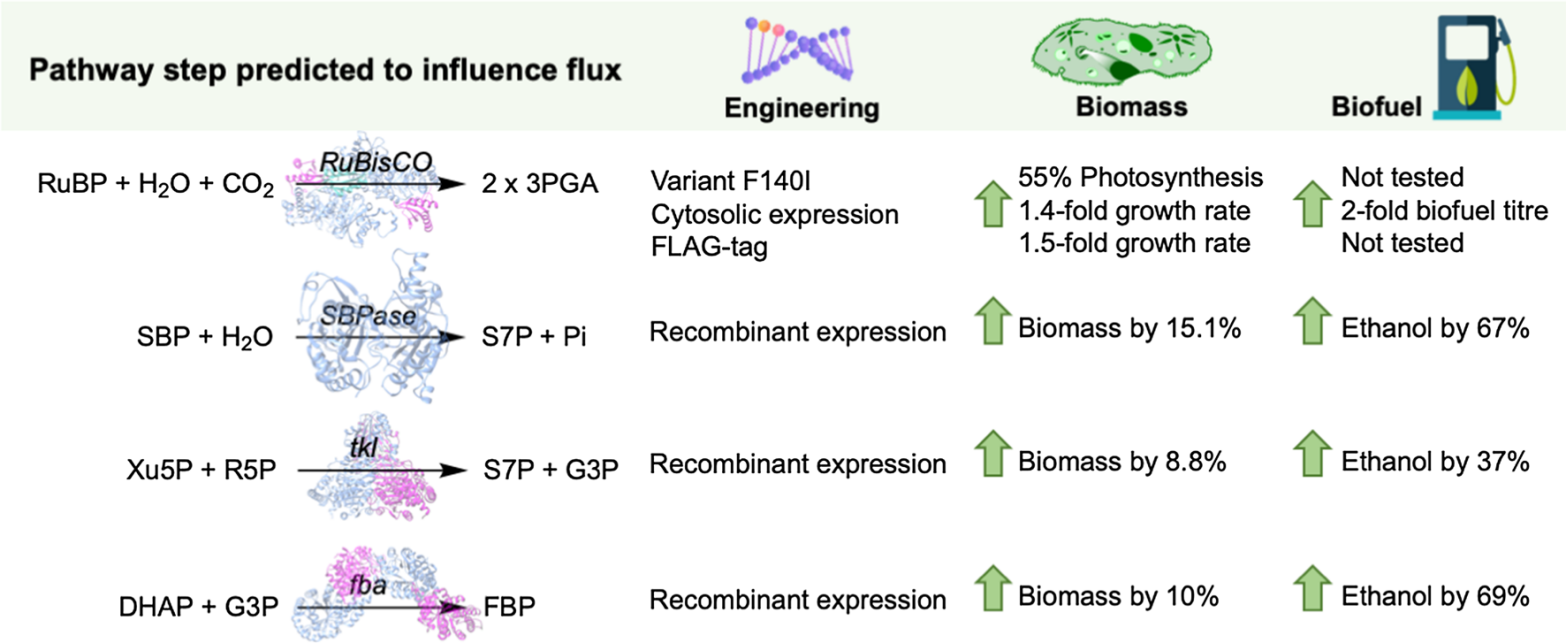
CBB pathway enzymatic steps predicted to positively influence flux control [31, 70, 72, 73, 77, 78]. Pathway intermediates: DHAP, dihydroxyacetone phosphate; FBP, fructose-1,6-bisphosphate; G3P, glyceraldehyde-3-phosphate; Pi, pyrophosphate; S7P, sedoheptulose-7-phosphate; SBP, sedoheptulose-1,7-bisphosphate; Xu5P, xylulose-5-phosphate. Enzymes/genes: *fba*, fructose-bisphosphate aldolase; *SBPase*, sedoheptulose-1,7-bisphosphatase; *tkl*, transketolase. The remaining pathway intermediates and enzymes are defined in Fig. 1 legend. The crystal structures of RuBisCO, SBPase, tkl and fba were generated in Chimera [79] using pdb accession codes 6hbc, 3oi7, 1trk and 1rv8, respectively (https://www.rcsb.org/)

Pyruvate supply has been identified as a limiting factor for ethanol production in *Synechocystis*, most likely due to insufficient carbon flux [30, 56, 57, 61]. Routes to similar products currently limited by acetyl-CoA availability have been reviewed by Choi et al. [62], where they discuss using shifting environmental conditions to improve titres through acetyl-CoA modulation. Pyruvate supply limitation was demonstrated by feeding exogenously supplied pyruvate (25 mM; with 50 mM NaHCO_3_) to the double integrated *Syn*-YQ4 strain, which increased both culture density and ethanol titres [30]. Since pyruvate is separated from carbon dioxide by only four reactions (Fig. 1), strengthening the CBB cycle could potentially increase the flux towards pyruvate. Central to this process is the carbon fixing enzyme RuBisCO, which is housed within carboxysomes that function to increase the localised carbon dioxide concentration around RuBisCO [63–65]. This enzyme is surprisingly inefficient and has long been earmarked for directed evolution to improve its performance. Unfortunately, attempts so far have been largely unsuccessful, with RuBisCO’s performance thought to be constrained due to the complexity of its reactive site chemistry [66–68]. However, the recent discovery of a highly active and assembly competent Form II RuBisCO from an unknown endosymbiont of a deep-sea tubeworm *Riftia pachyptila* shows promise as an alternative enzyme for recombinant expression in cyanobacteria [69]. Heterologous expression of this enzyme does not require the co-expression of chaperonins and it exhibits a 51% higher carboxylation efficiency than the RuBisCO from *Syn*-7002, when expressed in the heterotroph *E. coli* [69]. Alternatively, modified RuBisCO variants have been generated with enhanced carboxylation efficiencies and substrate specificities [70, 71]. For example, variant F140I in the large RuBisCO subunit had 2.9-fold improved carboxylation efficiency, which when expressed in *Syn*-6803 led to a 55% improvement in the photosynthetic rate (Fig. 2) [70]. This mutation is localised at the N- and C-terminal domain interface within each subunit. It is thought this mutation to isoleucine could result in an increased structural flexibility, which in turn may enhance catalytic turnover. Interestingly, this equivalent residue in plants and red/green algae is normally a conserved isoleucine or leucine, so it is unknown why cyanobacteria have phenylalanine in this position [70]. Unfortunately, the improved RuBisCO variants have not been tested in ethanologenic strains of *Synechocystis*, but these encouraging results suggest this may be a viable approach for improving carbon flux towards ethanol production.

Another approach to increasing the photosynthetic rate is to increase the expression of RuBisCO. This was performed by expressing two copies of RuBisCO within an isobutyraldehyde-producing *Syn*-7942 strain [72]. In vivo RuBisCO activity was increased 1.4-fold, with a concomitant two-fold increase in total isobutyraldehyde production after 7 days [72]. An alternative approach was the overexpression of RuBisCO within *Syn*-6803 with a FLAG tag on the large subunit. This strain showed a 1.52-fold increase in RuBisCO activity and an improved growth rate [73]. However, heterogeneously expressed RuBisCO localises to the cytosol [74], unless the correct targeting sequence is included. Therefore, it does not benefit from carbon dioxide accumulation within the carboxysomes, but it still shows an overall increase in the photosynthetic rate of *Synechocystis* [73, 74]. The latter may be in part due to an increased NADPH cycling between the photosynthetic machinery and the CBB cycle. If this is true, RuBisCO overexpression could enhance both carbon fixation and light-dependent photosynthesis, allowing cyanobacterial chassis to fix more carbon.

Looking beyond RuBisCO, other enzymes involved in pyruvate biosynthesis could be targeted. This includes the three-step pathway from the CBB intermediate 3-phosphoglycerate (3-PGA) to pyruvate catalysed by 2,3-bisphosphoglycerate-independent phosphoglycerate mutase (pgm), enolase (eno) and pyruvate kinase (pyk; Fig. 1). Increasing pyk activity may improve flux through pyruvate as intracellular levels of its substrate phosphoenolpyruvate (PEP) are ten-fold higher than pyruvate (Fig. 1; [75]). This was investigated using a lactic acid-producing *Syn*-6803 [58] and 2,3-butanediol (23BD)-producing *Syn*-7942 strains [54]. Overexpression of pyk generated ~ 135 mg/L 23BD, but the culture displayed a marked growth defect. Within these strains, overexpression of phosphoglycerate mutase (pgm) also increased flux towards pyruvate and carbon partitioning to 2,3-BD, whilst overexpression of *eno* only increased 2,3-BD titres (Fig. 1; [54].) Recent work identified a novel mechanism of pgm regulation by the Sll0944 gene product (PirC) which is under the control of the nitrogen regulator PII. Deletion of this pgm inhibitor increases the flux towards poly-β-hydroxybutyrate (PHB) and could conceivably direct this same additional flux to ethanol in a PHB deficient knockout strain [76].

A more extensive bioinformatics analysis was performed by generating a kinetic model of the entire CBB cycle using a random sampling of steady-state metabolite concentrations and enzyme kinetic parameters [77]. This analysis showed that the CBB cycle had an overall high intrinsic stability, as long as the metabolite ribulose 1,5-bisphosphate (RuBP) did not accumulate within the cell (Fig. 1). The CBB pathway enzymes that were predicted to exert a weak positive effect on overall network flux were sedoheptulose 1,7-bisphosphatase/fructose 1,6-bisphosphatase (SBPase), fructose-bisphosphate aldolase (fba), and transketolase (tkl; Fig. 2) [77].

The recombinant expression of CBB pathway enzymes, other than RuBisCO, in *Synechocystis* strains has been performed to assess the impact on ethanol production. For example, the overexpression of recombinant SBPase, tkl and fba all showed increased ethanol titres by 67%, 37% and 69%, respectively, when compared to a control strain (Fig. 2; [78]). In addition, carbon fixation rates increased, with total biomass production (dry cell weight and ethanol) increasing by 7.7%, 15.1%, 8.8% and 10.1%, respectively [78]. The tandem overexpression of fba and tkl yielded nine-fold and four-fold higher ethanol production than solely overexpressing fba or tkl, respectively [31]. However, the best performing fba/tkl strain only produced 1.2 g/L ethanol after 20 days [31], far below best performing *Synechocystis* strain after 26 days (5.50 gL^−1^ ethanol; [24]). These studies suggest overexpressing key enzymes involved in pyruvate production could improve overall flux towards ethanol production [31].

### Modification of competing or synergistic pathways

**Fig. 3:**
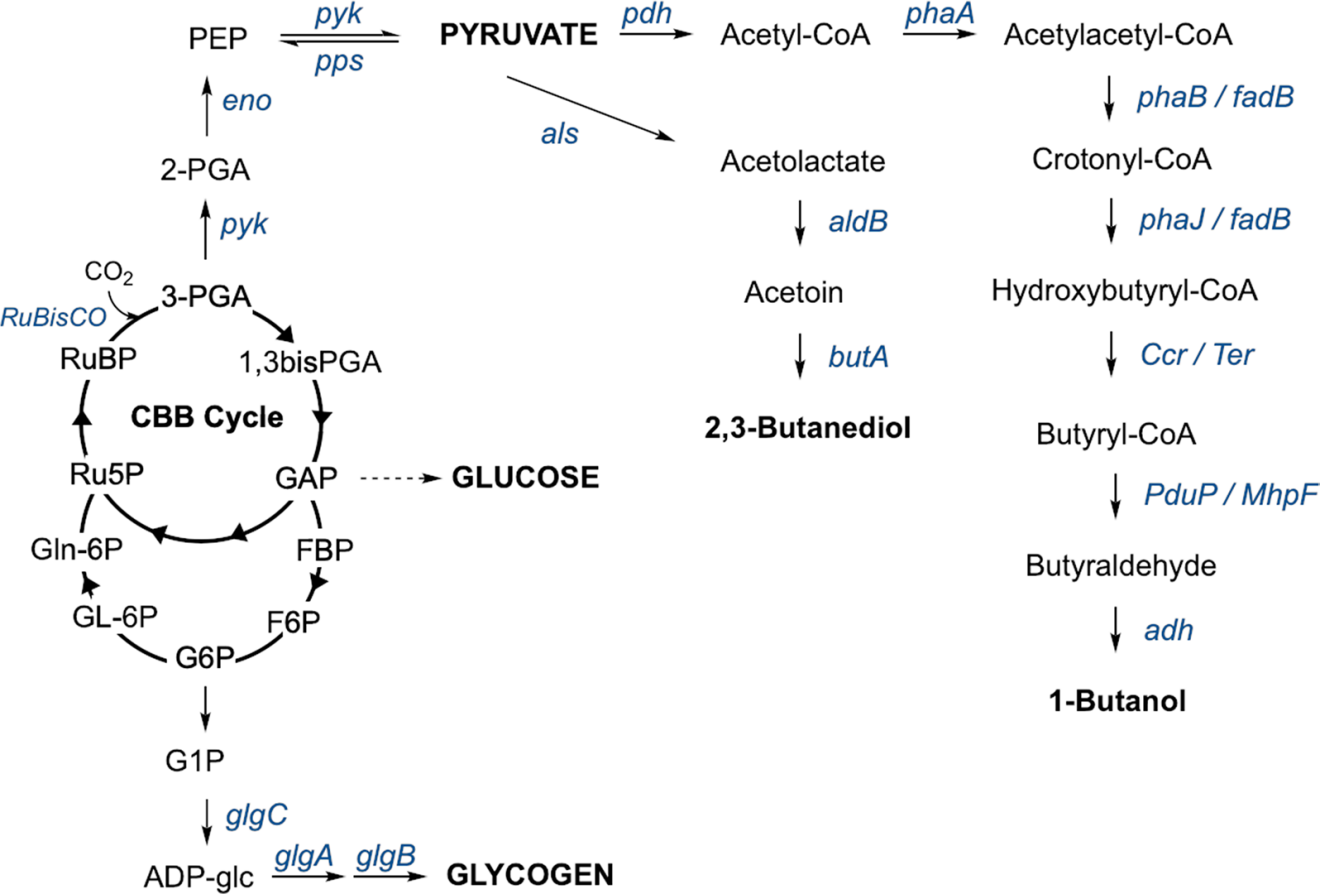
Metabolic pathways from the CBB cycle towards pyruvate and glycogen production. The enzymes are shown in blue. Pathway intermediates: ADP-glc, ADP-glucose; 1,3bisPGA, 1,3-bisphosphoglycerate; *aldB*, α-acetolactate decarboxylase; *als*, acetolactate synthase; *butA*, butanediol dehydrogenase; F6P, fructose-6-phosphate; Gln-6P, 6-phosphogluconate; GL-6P, 6-phosphogluconolactone; G1P, glucose-1-phosphate; G6P, glucose-6-phosphate; Ru5P, ribulose-5-phosphate. Enzymes/genes: *ccr*, crotonyl-CoA carboxylase/reductase; *glgA*, glycogen synthase; *glgB*, glycogen branching enzyme; *glgC*, ADP-glucose pyrophosphorylase; *PduP*/*MhpF*, acetaldehyde dehydrogenase; *phaJ*/*fadB*, enoyl-CoA hydratase; *Ter*, *trans*-2-enoyl-CoA reductase

Another approach to increasing pyruvate supply is to either knock out or knock down pathways that compete for pyruvate supply. Upregulating beneficial pathways, such as increasing acetate production, could improve titres of ethanol [80]. However, this is only practical if acetate can be efficiently converted into acetaldehyde by the action of an aldehyde dehydrogenase. One study looked at the effect of knocking-out phosphoenolpyruvate synthase (pps), which catalyses the production of PEP from pyruvate, the reverse direction of the reaction catalysed by pyk (Fig. 3). Knockout of *pps* was performed by the integration of an ethanologenic cassette at *slr0301* in *Syn*-6803 [45]. This led to a 41% increase in ethanol production to titres of 2.79 g/dry cell weight (DCW). Encouragingly, no growth defects were observed when the *pps* knockout was grown under constant light [45]. Interestingly, both pdc and adh transcripts in the *pps* knockout were 1.6-fold and 2.26-fold higher, respectively, compared to *Syn*-6803 strains with the ethanologenic cassette integrated into a neutral site [45]. This suggests that the higher pdc abundance, rather than an increased flux to pyruvate, may have been responsible for the observed ethanol titre improvements.

Another target gene responsible for flux away from pyruvate is phosphoenolpyruvate carboxylase [81] (ppc), which converts phosphoenolpyruvate into the TCA cycle intermediate oxaloacetate and malate (Fig. 1; [58]). A knockout of this gene in a recombinant *Syn*-6803 strain showed increased lactic acid production [58]. However, no quantification of the intracellular pyruvate pools was conducted, so there is only indirect evidence of the efficacy of this approach. Ppc is known to be part of a pathway that efficiently converts phosphoenolpyruvate into pyruvate via oxaloacetate and malate (Fig. 1)*.* This highlights the complexity of pyruvate core metabolism and its global metabolic implications [82]. Circumventing this complexity by knockouts in non-core pathways and modulating flux via environmental conditions may be a better alternative. Potential targets not involved in core pathways include knockouts/knockdowns of lactate dehydrogenase (*ldh*) and acetyl-CoA carboxylases (*acc*), to downregulate the production of lactate and fatty acid pathways, respectively (Fig. 1).

Major carbon storage mechanisms of *Synechocystis*, such as glycogen and (PHB) production, could be targeted to reduce the pyruvate flux towards these competing carbon sinks. Glycogen is a major carbon storage molecule that accumulates during daylight hours and is metabolised under dark conditions to provide energy for cellular survival [83–85]. *Synechocystis* glycogen content reaches around 22.7% of the DCW [86] and is thought to buffer against the presence of excess carbon [84]. The role of PHB in *Synechocystis* is less clear, but is thought to act as either a carbon reserve and/or serve as a redox sink when the reducing equivalents NAD(P)H are in excess [87, 88]. Under continuous culture conditions glycogen and PHB may play only minor roles, so knocking down key gene(s) in their biosynthesis may assist in increasing intracellular pyruvate levels or flux towards ethanol.

The deletion of both glycogen synthase genes (*glgA*; Fig. 3) in *Syn*-7002 was shown to reduce cellular glycogen abundance by 95.8%, leading to a significant increase in soluble sugar content [89]. A second study knocked out the two *glgA* genes by integrating two copies of the pdc_*Zm*_ + adh_*Syn*_ cassette [37]. As expected, this knockout severely impeded growth under diurnal conditions. However, no growth perturbations were observed under constant illumination, when carbon storage mechanisms are less critical [37, 84, 90, 91]. Ethanol titres reached 2.2 g/L after 10 days (0.22 g/L/day) under constant illumination; however, no control strain data were available with the ethanologenic cassette integrated into a neutral site. Therefore, it is not known how much the *glgA* knockouts contributed towards increasing ethanol titres [37].

The enzyme glucose-1-phosphate adenylyltransferase (glgC) is essential in the biosynthesis of glycogen by synthesising the intermediate ADP glucose. Knockouts of this gene in *Syn*-7942 show a 28% loss in carbon fixation compared to wild-type strains [92]. However, incorporation of an isobutanol production pathway into this Δ*glgC* variant rescued the total carbon fixation rates to wild-type levels, with 52% of the total carbon redirected into isobutanol biosynthesis. These isobutanol titres were 2.5-fold higher than for the glgC-containing control strain expressing the same isobutanol production cassette [92].

Under conditions of nitrogen starvation, the equivalent Δ*glgC* variant of *Syn*-6803 showed an accumulation of PHB, an alternative carbon sink [27]. Conversely, a glycogen (*glgC*/*glgA*)-overexpressing *Syn*-6803 strain with a disruption of PHB biosynthesis (acetyl-CoA acetyltransferase; *phaA*) showed a 13.7% increase in cellular glycogen, acetate, and succinate pools [93]. Therefore, knockouts of one or more of the PHB pathway genes could be another route to increase flux towards bioethanol production. This was tested by generating *Syn-*6803 strains where one of the two pdc_*Zm*_/adh_*Syn*_ ethanologenic cassettes was integrated into a neutral site (*slr0168* or *psbA2*) and the other at the *phaA* loci, (*Syn*-HZ24 and UL030, respectively) [24, 29]. The *Syn*-HZ24 strain generated ethanol titres around 5.5 g/L, but showed no difference between the PHB intact and knockout variants [24]. PHB does not represent a large carbon sink in *Syn-*6803, as no PHB was detectable when grown in normal cultivation media, and only 4.1% PHB per DCW was observed under nitrogen starved conditions [27, 94]. Therefore, targeting PHB knockouts alone is not likely to have as great an influence on increasing flux towards ethanol compared to impairing glycogen biosynthesis.

An ethanol-producing strain of *Syn*-6803 was generated containing knockouts of both the glycogen and PHB biosynthesis pathways (Δ*glgC*Δ*phaCE*/EtOH) to observe if there was a synergistic effect by impairing two carbon sinks [27]. This strain generated ethanol at a rate of 240 mg/g DCW/day under the nitrogen starvation conditions. This is an increase of ethanol titres by 11.8% from 0.297 g/L (Δ*glgC*) to 0.332 g/L (Δ*glgC*Δ*phaCE*) after 72 h [27]. Interestingly, in a high cell density culture (OD_730nm_ = 50), ethanol production rates were 1.08 and 2.01 g/L/day under light conditions of 40 and 80 µmol/m^2^/s, respectively [27]. Alternative approaches could be employed using conditions designed to increase glycogen stores, then rapidly converting them to ethanol. A genetic knockout of the acetate kinase ackA and overexpression of the RNA polymerase sigma factor sigE were recently shown to increase succinate production from glycogen under dark fermentative conditions by the modulation of flux towards PEP [95].

These studies suggest that substituting native cyanobacterial carbon sinks for alternative pathways can alter the carbon partitioning ratio in favour of biofuel production, leading to higher titres without the usual deficit in growth and carbon fixation rates. By effectively replacing the natural carbon sinks with the production of secretable ‘carbon stores’, the cells will naturally shift carbon partitioning towards these alternative pathways during times of high nutrient and light availability. These approaches may yield the greatest titre if applied to faster growing cyanobacterial strains, such as *Synechococcus elongatus* UTEX 2973 [96] or *Synechococcus sp*. PCC 11901 [97].

### Ethanol tolerance

Increasing the ethanol tolerance of cyanobacteria is an essential component of any strategy designed for enhancing ethanol production. Without increased tolerances, ethanol titres will be limited by what the host can withstand before metabolic output is decreased. This could lead to a productivity decrease or cellular death at ethanol titres below what is needed for an economically feasible process. The ethanol tolerances of cyanobacteria recorded to date are ~ 1.5% (w/v) [98], far below the required titre for effective recovery. Efforts have already been made to improve tolerance by engineering and overexpressing transporters [99] and performing adaptive evolution [98, 100].

## Environmental stress

**Fig. 4:**
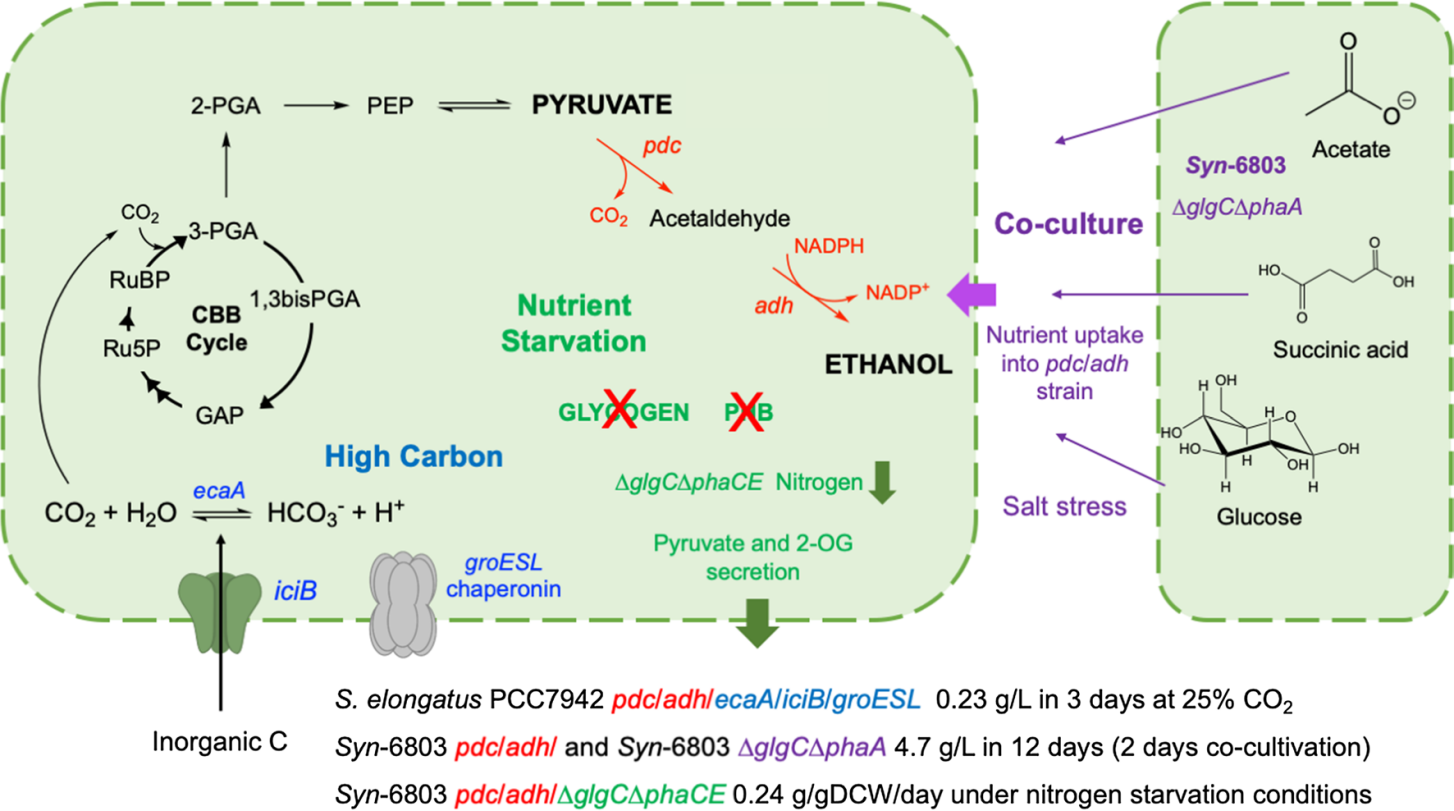
Effect of cyanobacterial chassis engineering and environmental stress responses on bioethanol production [27, 36, 39]. The enzymes/genes are colour coded to reflect which of the three engineered chassis they belong to. Enzymes/genes: *ecaA*, α-type carbonic anhydrase; *groESL*, alcohol tolerant chaperonin; *iciB*, inorganic carbon transporter. The remaining intermediates and enzymes are defined in Figs. 1, 2, 3 legends

Cyanobacterial growth and secondary metabolite production are inextricably linked to external factors, such as nutrient availability (minerals and carbon dioxide) and light access. The introduction of environmental stress, where one or more of the optimal growth conditions are perturbed, can lead to changes in metabolic flux and growth rate. Salt stress, nitrogen deficiency, high carbon availability, high and chromatic light stresses have all been shown to induce wholesale changes in cyanobacterial metabolism (Fig. 4). If these changes lead to the rerouting of key carbon metabolites towards pyruvate accumulation, higher titres of bioethanol could potentially be achieved. Therefore, the addition of carefully timed environmental stresses may be a complementary approach in addition to the genetic manipulation of cyanobacterial metabolic pathways.

### High carbon availability

Whilst cyanobacteria are evolved to atmospheric carbon dioxide levels (~ 0.04% v/v), enriched sparging at 1–5% CO_2_ (v/v) increases cyanobacterial growth [101]. Given the global concerns about carbon dioxide levels, the ‘recycling’ of heavy industry waste exhaust gases into bioethanol production could decrease its overall carbon footprint. Carbon supplementation can also be sourced from inorganic sodium (hydrogen) carbonate in conjunction with carbon capture technology from flue gases [102, 103]. Unfortunately, high levels of carbon dioxide as well as sulphur oxides from flue gases can acidify cyanobacterial culture media and perturb growth [104]. However, carbon supplementation in the form of bicarbonate can also act as a buffer against pH changes.

Cyanobacteria excrete exopolymeric carbohydrate substances, such as mannose, as part of a high carbon stress response [105–108]. This has been reported in *Syn*-6803 and *Syn*-7002 strains overexpressing bicarbonate importers [105, 106], and when cyanobacteria are grown in high carbon environments [105, 107, 108]. Cultivation of *Syn*-7002 with 1% CO_2_ at 600 μmol photons/m^2^/s light exposure led to a 31% cellular glycogen content, compared to just 9.4% with atmospheric carbon dioxide [18]. The tendency to release excess assimilated carbon as an overflow product could be exploited by introducing an ethanologenic sink, funnelling this excess carbon into secondary product formation.

Adaptation of cyanobacteria to thrive under high (5–25%) carbon dioxide conditions can be achieved by co-overexpressing a putative inorganic carbon transporter (*ictB*) and an extracellular α-type carbonic anhydrase (*ecaA*) [39]. The latter gene catalyses the interconversion of carbon dioxide and water with carbonic acid/bicarbonate. This was examined with a *Syn*-7942 strain, which also contained the pdc_*Zm*_/adh_*Zm*_ cassette and a recombinant alcohol tolerance gene *groESL* [39]. Ethanol titres reached ~ 0.23 g/L in this strain after 3 days sparging at 25% CO_2_ (Fig. 4) [39].

An alternative strategy to improve cyanobacterial tolerance to high carbon loading is to screen variants generated by random mutagenesis for desirable traits, similar to the study with *Chlorella vulgaris* ESP-31 [109]. In this case, two carbon dioxide- and thermo-tolerant *C. vulgaris* variants were obtained from methyl‑*N*'‑nitro‑*N*‑nitrosoguanidine random mutagenesis. Higher photosynthetic activity and biomass productivity were obtained at higher temperatures (40 °C; 25% CO_2_) with increased tolerance to flue gas contaminants (80–90 ppm SO_2_; 90–100 ppm NO). In addition, higher levels of carbohydrate and lipid contents were detected compared to wild-type ESP-31 strain [109].

In the case of high carbon dioxide sparging, optimisation is needed as perpetually high (25%) carbon dioxide levels reduce cyanobacterial growth compared to lower concentrations (5%) [39], which could reduce overall ethanol productivity of the system. Higher carbon dioxide concentrations could be used when cells have grown to stationary phase to induce a carbon shocked state at a higher cell density. Coupling this environmental stress with a knockout of glycogen synthesis and/or cycling between high and low CO_2_ levels would be particularly interesting as glycogen acts as a carbon buffer and could therefore direct more carbon towards ethanol synthesis.

### Salt stress

Exposure of cyanobacteria to high salt solutions induces the synthesis of osmolytes, which protects the cell from dehydration [110, 111]. Typical osmolytes include sucrose, trehalose, glucosyl-glycerol and glycine betaine. This adaption changes the carbohydrate profile of cells by redistributing carbon from insoluble to soluble forms, which could potentially increase carbon flux towards pyruvate and ethanol production [112]. For example, a sodium stress cycling method was developed in the salt tolerant cyanobacteria, *Arthrospira (Spirulina) maxima*. This relied on an initial high salt growth phase to accumulate sugars, followed by hypoionic stress (low salt shock) to force the catabolism of stored carbon during autofermentation [112]. This latter step leads to the production of large amounts of NAD(P)H. To mitigate against this, the endogenous ethanol-producing pathway from acetyl-CoA to ethanol was activated which re-oxidises four NAD(P)H molecules [112]. This resulted in ethanol titres of 0.0346 g/gDCW/day, a 121-fold increase over cells grown exclusively under low salt conditions [112].

To the best of our knowledge, sodium stress cycling has not been performed in *Syn*-6803, *Syn*-7002 or *Syn*-7942. In the case of *Syn*-7942, the accumulation of the osmoprotectant sucrose has been greatly enhanced by the overexpression of sucrose exporter *cscB* in cells grown at 200 mM NaCl, which yielded sucrose at levels up to 80% of the cell biomass [55]. This represents a large pool of carbon that could potentially be catabolised and redirected towards pyruvate in a manner similar to the autofermentation method [112]. Excess NAD(P)H generated under autofermentation could be utilised by adh to improve ethanol titres. Since PHB synthesis has recently been touted as an NAD(P)H sink [87, 88, 113], the use of a sodium stress cycling along with knockouts of PHB synthesis and NADH-dependent lactate dehydrogenase [114] would make a compelling case to study how excess NAD(P)H and osmolytes are used in *Syn-*6803 and *Syn*-7942 strains.

The combinatorial effect of culturing a pdc_*Cv*_/adh_*Syn*_ engineered *Syn-*6803 strain in high salt media has been investigated; however, they did not employ sodium salt stressing [36]. Cultivation with 50–150 mM NaCl led to ethanol productivities and cell counts similar to a no salt control, with higher salt concentrations leading to decreases in ethanol titres and biomass accumulation. This suggests that high salt stress alone does not increase ethanol production, as the osmoprotectants accumulated would likely be maintained by the cell during constant salt stress conditions [36]. In contrast, prolonged salt stress has been successful at increasing isobutanol production in *Syn*-7942. Growth in 2% sea salt media inhibits CBB cycle activity, whilst enhancing lipid degradation, which increased the cellular NADH pool 5.1-fold [115]. This enlarged NADH pool was utilised by the heterologously expressed adh from *Lactococcus lactis*, which produced 0.637 g/L within 20 days, a five-fold increase over cultivation under low salt conditions [115].

### Nutrient starvation

It has been well characterised that nitrogen, phosphorus and sulphur deprivation can induce wholesale changes in cyanobacterial metabolism [116]. For example, nitrogen starvation can induce the accumulation of both glycogen and PHB [27, 113, 117], as glycogen turnover products accumulate as PHB [113]. Nitrogen starvation is one stress known to increase pyruvate concentration [81]. Studies have documented that in *Syn-*6803, a combination of nitrogen starvation and deletion of glycogen and PHB synthesis genes leads to a redistribution of carbon flux towards pyruvate and α-ketoglutarate (αKG) [27, 84, 118]. This leads to the export of pyruvate and αKG from the cell in large quantities (up to 0.0404 gL^−1^ and 0.0705 gL^−1^ respectively) after 48 h [27, 84, 118]. This has been described as an overflow of metabolism or a ‘spill over’ of partially oxidised carbonic acids. It is a means of dissipating excess ATP non-productively when the catabolism of an energy source (carbohydrate) is in excess of the anabolic utilisation rates for growth and cell maintenance [119].

This overflow metabolism has been exploited in *Syn-*6803 by the introduction of an ethanologenic pathway into variants deficient in both glycogen and PHA synthesis (Δ*glgC*Δ*phaCE*/EtOH; Fig. 4), as described earlier [27]. Ethanol production was incorporated to act as a new carbon sink to replace glycogen and PHB synthesis and avoid the wasteful excretion of pyruvate and αKG. Cultivation under nitrogen-limited conditions led to a 2.8-fold increase in ethanol productivity. In contrast, cultivation of *Syn-*6803 under nitrogen starvation conditions with intact glycogen and PHB pathways showed a 0.65-fold decrease in ethanol production (0.0309 g/DCW/d) [27]. This suggests that the absence of major carbon sink pathways is required before the beneficial effects of nitrogen starvation on ethanol production are observed.

The addition of an ethanologenic pathway to *Syn-*6803 Δ*glgC*Δ*phaCE* strains is not sufficient to completely eliminate metabolic overflow, as around 20% of fixed carbon is still being secreted as αKG under nitrogen starvation conditions [27]. Therefore, further engineering is required to shift carbon flux from αKG to pyruvate, enabling ethanol production to be a more efficient carbon sink. This could potentially be done via the TCA cycle, with the intermediate malate being converted into pyruvate via the overexpression of an NAD(P)H-dependent malic enzyme (me; Fig. 1). Overall, whilst total carbon fixation and photosynthetic efficiency were reduced with *Syn-*6803 Δ*glgC*Δ*phaCE*/EtOH variants under nitrogen starvation, improved ethanol yields were obtained. This demonstrates higher carbon fixation rates may not be necessary to increase the overall flux towards pyruvate (and ultimately ethanol).

Pyruvate is the immediate precursor to the engineered pdc_Zm_/adh_*Syn*_ ethanol pathway, so it was not surprising that the presence of exogenously supplied pyruvate enhanced ethanol titres in *Syn*-YQ4 [30]. As nitrogen limitation-dependent metabolic spill over causes pyruvate secretion into the culture medium [27, 84, 118, 119], this presents an interesting opportunity to partition the pyruvate secretion and ethanol production functionalities into two separate microorganisms, creating a co-culture strategy [30, 36, 54].

### Impact of excess light

Light intensity is positively correlated with both the number of β-carboxysomes per cell and the carbon fixation capacity [120], and is essential for maximising biomass accumulation [121–123]. However, cyanobacteria are known to experience light-induced stress dependent on the light intensity [121] and wavelength [124]. A common mechanism of adaptation to changes in light intensity is the alteration of flux between the synthesis and use of glycogen and other carbon sinks [125]. The glycogen-deficient *Syn*-6803 variant Δ*glgC* under nitrogen starvation is known to excrete organic acids, mainly pyruvate and 2-oxoglutarate [118]. However, studies have shown that under high light conditions this Δ*glgC* variant shows an increased ATP/ADP ratio leading to a metabolic overflow of organic acids [125]. These conditions were not tested with an ethanologenic strain; however, a substitution of nitrogen starvation for high illumination conditions could be a potential route towards increasing ethanol titres in *Syn-*6803 Δ*glgC*Δ*phaCE*/EtOH strains.

These high light stress-induced changes could potentially be paired with other gene deletions to direct a metabolic overflow towards ethanol production. Recent computational models have been generated to identify genetic knockouts for improving ethanol production [44, 126]. However, these models are based on values for light exposure, carbon uptake, ATP flux and NAD(P)/NAD(P)H turnover that would occur under standard growth conditions [44, 126]. As these parameters would be different under high light stress, the predicted combination of gene deletions for higher ethanol titres may not be a suitable solution under high light stress conditions [126]. For example, a genome‐scale metabolic network model for *Syn*-6803 was generated that predicted five target genes for deletion to increase ethanol titres [44]. This included a simulation of knocking out acetyl-CoA synthetase and the putative acetate transporter, which could redirect carbon leaking as ‘acetate overflow’, leading to increased ethanol production [44]. This mimics an observed metabolic overflow of organic acids induced by high light stress [125]. Whilst an understanding of the impact of light stress on ethanologenic cyanobacteria is limited, there is potential for this environmental stress to be employed as an additional tool in optimising ethanol production in cyanobacteria, when coupled to genetic deletions for accumulating fixed carbon.

### Co-cultivation

Co-cultivation strategies centred around metabolic overflow and environmental stress could be united to enhance ethanol production. For example, a non-ethanologenic strain is used to secrete metabolic precursors, which are used by a second strain for ethanol production. Environmental stresses and genetic deletions are used to enable metabolic overflow and precursor secretion in support of ethanol production.

Co-cultivation of two variant strains of *Syn*-6803 illustrates this approach. One variant strain was deficient in glycogen and PHA biosynthesis (Δ*glgC*Δ*phaA*), whilst the other expressed the ethanologenic cassette (*pdc*/*adh*; Fig. 4) [36]. Glucose, acetate and succinate secretion by the Δ*glgC*Δ*phaA* strain was enabled during growth under high salt conditions. These metabolites were then subsequently utilised by the *pdc*/*adh*-containing variant strain, which generated 4.7 g/L ethanol under high salt stress conditions [36]. Co-cultivation under these conditions showed an overall improvement in the titres of ethanol by the *pdc*/*adh* strain, in spite of a decrease in the overall biomass production compared to a mono-culture.

Other environmental stress and/or knockout combinations that cause metabolic overflow could in principle increase ethanol titres, but remain unexplored. These include glycogen knockout mutants under high light stress [124, 125] and nitrogen limitation [118], and glycogen/PHB knockout strains under nitrogen limitation conditions [27, 84, 118]. High carbon stress environments [105–108] could be paired with glycogen knockout mutants to enhance the secretion of exopolymeric carbohydrate substances.

For co-cultivation strategies to become economically feasible, it may be necessary to use faster growing cyanobacterial species, such as *Synechococcus elongatus* UTEX 2973 [96] or *Synechococcus sp*. PCC 11901 [97] to supply extracellular pyruvate and/or αKG to the ethanologenic strain. High ethanol yielding organisms, such as *S. cerevisiae,* could also be used, provided suitable co-cultivation conditions are devised. *Synechococcus sp*. PCC 11901 (*Syn*-11901) is highly salt tolerant (up to 10% (w/v) NaCl): co-cultivation of potential *Syn*-11901 strains with glycogen and/or PHB knockouts might be possible in combination with halophilic heterotrophs (e.g. *Halomonas* species). The use of high salt concentrations during growth would reduce microbial contamination, reducing the costs associated with sterilisation and maintenance of aseptic conditions in conventional industrial-scale fermentations [127].

### Feedstock engineering

The use of microalgae for producing high value products has been reviewed (e.g. [23]), but there has been less information and comment on how cyanobacteria could be used as a microbial feedstock for secondary product generation. Hydrolysis of cyanobacterial biomass would yield a rich carbon feedstock for heterotrophic production organisms. As an example, *Syn*-6803 cells can act as a carbon source for biopropane production using an engineered strain of *Halomonas* [127]. Similarly, hydrolysed *Syn*-7942 cells engineered for high carbohydrate content (bacterial cellulose) have been used as a carbon and energy source with *Z. mobilis*, generating 7.2 g/L ethanol with 91% theoretical yield [128]. Ethanol-producing strains could also be recycled after ethanol generation as a feedstock material in a modular bioprocessing plant [127, 129].

Salt stress conditions increase sucrose content in *Synechocystis* [55], whilst high carbon environments lead to exopolymeric carbohydrate secretion [105] as well as enhanced glycogen content [18]. Also, nitrogen starvation increases glycogen content [27]. Increased carbohydrate content of spent cyanobacterial cells after growth/ethanol production under environmental stresses could enhance the quality of the residual biomass as a feedstock. Ultimately, integrating feedstock engineering and synthetic biology for product formation will help close the loop in a circular bioeconomy.

## Conclusions

The journey towards the commercialisation of autotrophic bioethanol production is far from over, yet progress in ethanologenic cassette and chassis redesign has already increased ethanol titres by 24-fold [15, 24, 27]. Ethanol titres are currently 14-fold lower than the minimum required for the energy efficiency of ethanol recovery by distillation to become commercially viable, so step changes are needed, for this and other recovery methods [9, 130, 131]. Recent techno-economic analysis (TEA) of the viability of scaled cyanobacterial ethanol production suggested that the operational costs would be greater than the value of ethanol produced [9]. To achieve economic feasibility ethanol titres and/or value (price per kilogramme) must be significantly increased, or co-produced with a higher value second product [9]. A similar TEA analysis was performed for biopropane production in *Halomonas*, where the current titres are too low to enable the process to compete with low cost fossil fuel sources [127]. In the latter case, the co-production of higher value PHB and ectoine was proposed to increase the overall cost efficiency of the process and may tip the balance towards commercial viability. The enrichment of the carbohydrate content of *Synechocystis* biomass may improve the economic feasibility for downstream use as a microbial feedstock, verses current secondary uses as a general fertiliser [132].

Since the existing ethanologenic cassette is considered to be largely optimised, it may be preferable to focus on increasing the in vivo pyruvate supply by either upregulating pathway precursor enzymes or decreasing flux through competing routes [30]. The substitution of existing carbon sinks (glycogen and PHB) for an ethanologenic cassette could enable the decoupling the CBB cycle from growth and maintenance, and lead to increased carbon dioxide fixation and light capture steps. Optimisation of the carbon partitioning towards ethanol production in combination with nitrogen-limited metabolic overflow may help shift the balance in favour of increasing titres of ethanol as a secretable alternative carbon sink. As many competing pathways provide alternative carbon sinks or deplete the pyruvate pool, genetic knockouts can assist in reducing carbon flux away from ethanol production whilst maintaining relatively high viability and growth rate. In addition, the use of environmental stresses could be used to modify the carbohydrate profile and energy or redox homeostasis of cells, inducing wholesale changes in metabolism that gear up the cell for ethanol production. Such a combinatorial approach could result in the titre improvements needed to make ethanol recovery viable, the productivity improvements needed to make photosynthetic bioethanol economically viable, and bring about the advent of a new renewable source of bioethanol from sunlight and CO_2_.

## Data Availability

Not applicable.

## Abbreviations

Aas: Acyl-ACP synthetase
Acc: Acetyl-CoA carboxylase
Acs: Acetyl-CoA-synthase
AckA: Acetate kinase
Adh: Alcohol dehydrogenase
ADP-glc: ADP-glucose
AEF: Alternate electron flow
AlDH: Aldehyde dehydrogenase
2,3-BD: 2,3-Butanediol
CB cycle: Calvin-Benson-Bassham cycle
Chl a: Chlorophyll a
DCW: Dry cell weight
DHAP: Dihydroxyacetone phosphate
Eno: Enolase
fba: Fructose-bisphosphate aldolase
FBP: Fructose-1,6-bisphosphate
GlgA(1/2): Glycogen synthase (1/2)
GlgB: 1,4-α-Glucan branching enzyme
GlgC: Glucose-1-phosphate adenylyltransferase
G1P: Glucose-1-phosphate
G3P: Glyceraldehyde-3-phosphate
G6P: Glucose-6-phosphate
αKG: α-Ketoglutarate
Ldh: Lactate dehydrogenase
LEF: Linear electron flow
ME: Malic enzyme
OAA: Oxaloacetate
2-OG: 2-Oxoglutarate
PBS: Phycobilisomes
Pdc: Pyruvate decarboxylase
PEP: Phosphoenolpyruvate
2-PGA: 2-Phosphoglycerate
3-PGA: 3-Phosphoglycerate
Pgm: 2,3-Bisphosphoglycerate-independent phosphoglycerate mutase PhaA: acetyl-CoA acetyltransferase
PhaB: Acetoacetyl-CoA reductase
PhaC/E: Poly(3-hydroxyalkanoate) polymerase
PHB: Polyhydroxybutyrate
Pi: Pyrophosphate
Pps: Phosphoenolpyruvate synthase
PSI: Photosystem 1
PSII: Photosystem 2
Pta: Phosphate acetyltransferase
Pyk: Pyruvate kinase
PyrOR: Pyruvate oxidoreductase
RuBP: Ribulose biphosphate
RuBisCO: Ribulose biphosphate carboxylase/oxygenase
S7P: Sedoheptulose-7-phosphate
SBP: Sedoheptulose-1,7-bisphosphate
SBPase: Sedoheptulose-1,7-bisphosphatase
*Syn-*6803: *Synechocystis* sp. PCC 6803
*Syn*-7002: *Synechococcus* sp. PCC 7002
Syn-7942: *Synechococcus elongatus PCC 7942*
tkl: Transketolase
Xu5P: Xylulose-5-phosphate
YqhD: NADPH-dependent aldehyde reductase

## References

[1] Bugg TDH, Resch MG (2015). Editorial overview: Energy: prospects for fuels and chemicals from a biomass-based biorefinery using post-genomic chemical biology tools. Curr Opin Chem Biol.

[2] IPCC. Climate change 2021: The physical science basis. Contribution of working group I to the sixth assessment report of the intergovernmental panel on climate change. Masson-Delmotte V, Zhai P, Pirani A, Connors SL, Péan C, Berger S, Caud N, Chen Y, Goldfarb L, Gomis MI *et al*, Cambridge, UK; 2021. In Press.

[3] Angili TS, Grzesik K, Rödl A, Kaltschmitt M (2021). Life cycle assessment of bioethanol production: a review of feedstock, technology and methodology. Energies.

[4] International Renewable Energy Agency. Renewable energy prospects for the European Union. 2018. https://www.irena.org/publications/2018/Feb/Renewable-energy-prospects-for-the-EU. Accessed 28 Sept 2019.

[5] Al-Azkawi A, Elliston A, Al-Bahry S, Sivakumar N (2019). Waste paper to bioethanol: current and future prospective. Biofuels Bioprod Biorefin.

[6] U.S Department of Energy. Ethanol Fuel Basics. 2020. https://afdc.energy.gov/fuels/ethanol_fuel_basics.html. Accessed 18 May 21.

[7] Alalwan HA, Alminshid AH, Aljaafari HAS (2019). Promising evolution of biofuel generations Subject review. Renew Energy Focus.

[8] Johnson E (2009). Goodbye to carbon neutral: Getting biomass footprints right. Environ Impact Assess Rev.

[9] da Silva A, Brazinha C, Costa L, Caetano NS (2020). Techno-economic assessment of a *Synechocystis* based biorefinery through process optimization. Energy Rep..

[10] Hannon M, Gimpel J, Tran M, Rasala B, Mayfield S (2010). Biofuels from algae: challenges and potential. Biofuels Bioprod Biorefin.

[11] de Farias Silva CE, Gris B, Sforza E, Rocca NL, Bertucco A (2016). Effects of sodium bicarbonate on biomass and carbohydrate production in *Synechococcus* PCC 7002. Chem Eng Trans.

[12] Yoshikawa K, Toya Y, Shimizu H (2017). Metabolic engineering of *Synechocystis* sp. PCC 6803 for enhanced ethanol production based on flux balance analysis. Bioprocess Biosyst Eng..

[13] Ramey CJ, Barón-Sola Á, Aucoin HR, Boyle NR (2015). Genome engineering in cyanobacteria: where we are and where we need to go. ACS Synth Biol.

[14] Heyer H, Krumbein WE (1991). Excretion of fermentation products in dark and anaerobically incubated cyanobacteria. Arch Microbiol.

[15] Deng MD, Coleman JR (1999). Ethanol synthesis by genetic engineering in cyanobacteria. Appl Environ Microbiol.

[16] Dexter J, Armshaw P, Sheahan C, Pembroke JT (2015). The state of autotrophic ethanol production in Cyanobacteria. J Appl Microbiol.

[17] Zahra, Choo, Lee, Parveen. Cyanobacteria: Review of current potentials and applications. Environments. 2020;7:13.

[18] Aikawa S, Nishida A, Ho S-H, Chang J-S, Hasunuma T, Kondo A (2014). Glycogen production for biofuels by the euryhaline cyanobacteria *Synechococcus* sp. strain PCC 7002 from an oceanic environment. Biotechnol Biofuels..

[19] Rawat I, Ranjith Kumar R, Mutanda T, Bux F (2011). Dual role of microalgae: Phycoremediation of domestic wastewater and biomass production for sustainable biofuels production. Appl Energy.

[20] Oren A (2015). Cyanobacteria in hypersaline environments: biodiversity and physiological properties. Biodivers Conserv.

[21] Cheng J, Huang Y, Feng J, Sun J, Zhou J, Cen K (2013). Improving CO_2_ fixation efficiency by optimizing *Chlorella* PY-ZU1 culture conditions in sequential bioreactors. Bioresour Technol.

[22] Chittora D, Meena M, Barupal T, Swapnil P, Sharma K (2020). Cyanobacteria as a source of biofertilizers for sustainable agriculture. Biochem Biophys Rep..

[23] Chew KW, Yap JY, Show PL, Suan NH, Juan JC, Ling TC (2017). Microalgae biorefinery: High value products perspectives. Bioresour Technol.

[24] Gao Z, Zhao H, Li Z, Tan X, Lu X (2012). Photosynthetic production of ethanol from carbon dioxide in genetically engineered cyanobacteria. Energy Environ Sci.

[25] Yomano LP, York SW, Zhou S, Shanmugam KT, Ingram LO (2008). Re-engineering *Escherichia coli* for ethanol production. Biotechnol Lett.

[26] Ji H, Yu J, Zhang X, Tan T (2012). Characteristics of an immobilized yeast cell system using very high gravity for the fermentation of ethanol. Appl Biochem Biotechnol.

[27] Namakoshi K, Nakajima T, Yoshikawa K, Toya Y, Shimizu H (2016). Combinatorial deletions of glgC and phaCE enhance ethanol production in Synechocystis sp. PCC 6803. J Biotechnol..

[28] Armshaw P, Carey D, Quinn L, Sheahan C, Pembroke J. Optimisation of ethanol production in *Synechocystis* PCC 6803, the DEMA approach. In: 1st International Solar Fuels Conference (ISF-1): 2015; Uppsala, Sweden.

[29] da Silva T, Passarinho PC, Galriça R, Zenóglio A, Armshaw P, Pembroke JT (2018). Evaluation of the ethanol tolerance for wild and mutant *Synechocystis* strains by flow cytometry. Biotechnol Rep..

[30] Luan G, Qi Y, Wang M, Li Z, Duan Y, Tan X (2015). Combinatory strategy for characterizing and understanding the ethanol synthesis pathway in cyanobacteria cell factories. Biotechnol Biofuels.

[31] Roussou S, Albergati A, Liang F, Lindblad P (2021). Engineered cyanobacteria with additional overexpression of selected Calvin-Benson-Bassham enzymes show further increased ethanol production. Metab Eng Commun..

[32] Baier K, Germer F, Shi T, Duerhing U. Genetically enhanced cyanobacteria for the production of a first chemical compound harbouring Zn, Co or Ni -inducible promoters. 2013. Patent PCT/EP2012/076790 United States.

[33] Dienst D, Georg J, Abts T, Jakorew L, Kuchmina E, Börner T (2014). Transcriptomic response to prolonged ethanol production in the cyanobacterium *Synechocystis* sp. PCC6803. Biotechnol Biofuels..

[34] Dehring U, Kramer D, Ziegler K. Selection of ADH in genetically modified cyanobacteria for the production of ethanol 2009. Patent WO2013098267A1. United States.

[35] Dexter J, Fu P (2009). Metabolic engineering of cyanobacteria for ethanol production. Energy Environ Sci.

[36] Velmurugan R, Incharoensakdi A (2020). Co-cultivation of two engineered strains of *Synechocystis* sp. PCC 6803 results in improved bioethanol production. Renew Energy..

[37] Wang M, Luan G, Lu X (2020). Engineering ethanol production in a marine cyanobacterium *Synechococcus* sp. PCC7002 through simultaneously removing glycogen synthesis genes and introducing ethanolgenic cassettes. J Biotechnol..

[38] Reppas NB. Metabolic switch. 2012. Patent 13/304,034 United States.

[39] Chou H-H, Su H-Y, Chow T-J, Lee T-M, Cheng W-H, Chang J-S (2021). Engineering cyanobacteria with enhanced growth in simulated flue gases for high-yield bioethanol production. Biochem Eng J..

[40] Piven I, Friedrich A, Dühring U, Uliczka F, Baier K, Inaba M *et al.* Cyanobacterium sp. for production of compounds. 2015. Patent EP2935566A4. United States.

[41] Gundolf R, Oberleitner S, Richter J (2019). Evaluation of new genetic toolkits and their role for ethanol production in cyanobacteria. Energies.

[42] Dühring U, Baier K, Germer F, Shi T. Genetically enhanced cyanobacteria for the production of a first chemical compound harbouring Zn, Co or Ni -inducible promoters. 2017. Patent WO2013098267A1. United States.

[43] Cooley JW, Vermaas WFJ (2001). Succinate dehydrogenase and other respiratory pathways in thylakoid membranes of *Synechocystis* sp. strain PCC 6803: Capacity comparisons and physiological function. J Bacteriol..

[44] Lasry Testa R, Delpino C, Estrada V, Diaz SM (2019). In silico strategies to couple production of bioethanol with growth in cyanobacteria. Biotechnol Bioeng.

[45] Gao EB, Penglin Y, Xu Y, Yangjie Z, Kyere-Yeboah K, Chen G. Increased ethanol production by disrupting the competitive phosphoenolpyruvate synthesis pathway and enhancing the expression of ethanol-producing genes in *Synechocystis* sp. PCC 6803. 2020:Preprint 10.21203/rs.2.55/v2.

[46] Miao R, Liu X, Englund E, Lindberg P, Lindblad P (2017). Isobutanol production in *Synechocystis* PCC 6803 using heterologous and endogenous alcohol dehydrogenases. Metab Eng Commun.

[47] Luan G, Zhang S, Lu X (2020). Engineering cyanobacteria chassis cells toward more efficient photosynthesis. Curr Opin Biotechnol.

[48] Bartasun P, Prandi N, Storch M, Aknin Y, Bennett M, Palma A (2019). The effect of modulating the quantity of enzymes in a model ethanol pathway on metabolic flux in *Synechocystis* sp. PCC 6803. Peer J..

[49] Siegert P, McLeish MJ, Baumann M, Iding H, Kneen MM, Kenyon GL (2005). Exchanging the substrate specificities of pyruvate decarboxylase from *Zymomonas mobilis* and benzoylformate decarboxylase from *Pseudomonas putida*. Protein Eng Des Sel.

[50] Ruffing AM, Jensen TJ, Strickland LM (2016). Genetic tools for advancement of *Synechococcus* sp. PCC 7002 as a cyanobacterial chassis. Microb Cell Fact..

[51] Sengupta A, Madhu S, Wangikar PP. A library of tunable, portable, and inducer-free promoters derived from cyanobacteria. ACS Synth Biol. 2020;9:1790–801. 10.1021/acssynbio.0c00152 32551554

[52] Zhou J, Zhang H, Meng H, Zhu Y, Bao G, Zhang Y (2014). Discovery of a super-strong promoter enables efficient production of heterologous proteins in cyanobacteria. Sci Rep.

[53] Liu X, Xie H, Roussou S, Lindblad P (2022). Current advances in engineering cyanobacteria and their applications for photosynthetic butanol production. Curr Opin Biotechnol.

[54] Oliver JWK, Atsumi S (2015). A carbon sink pathway increases carbon productivity in cyanobacteria. Metab Eng.

[55] Ducat DC, Avelar-Rivas JA, Way JC, Silver PA (2012). Rerouting carbon flux to enhance photosynthetic productivity. Appl Environ Microbiol.

[56] Oliver JWK, Machado IMP, Yoneda H, Atsumi S (2013). Cyanobacterial conversion of carbon dioxide to 2,3-butanediol. Proc Natl Acad Sci USA.

[57] Kopka J, Schmidt S, Dethloff F, Pade N, Berendt S, Schottkowski M (2017). Systems analysis of ethanol production in the genetically engineered cyanobacterium *Synechococcus* sp. PCC 7002. Biotechnol Biofuels..

[58] Angermayr SA, van der Woude AD, Correddu D, Vreugdenhil A, Verrone V, Hellingwerf KJ (2014). Exploring metabolic engineering design principles for the photosynthetic production of lactic acid by *Synechocystis* sp. PCC6803. Biotechnol Biofuels..

[59] Miao R, Xie H, Liu X, Lindberg P, Lindblad P (2020). Current processes and future challenges of photoautotrophic production of acetyl-CoA-derived solar fuels and chemicals in cyanobacteria. Curr Opin Chem Biol.

[60] Martínez L, Redondas V, García A-I, Morán A (2011). Optimization of growth operational conditions for CO_2_ biofixation by native *Synechocystis* sp. J Chem Technol Biotechnol.

[61] Kanno M, Carroll A, Atsumi S (2017). Global metabolic rewiring for improved CO_2_ fixation and chemical production in cyanobacteria. Nat Commun.

[62] Choi YN, Lee JW, Kim JW, Park JM (2020). Acetyl-CoA-derived biofuel and biochemical production in cyanobacteria: a mini review. J Appl Phycol.

[63] Kaplan A, Badger MR, Berry JA (1980). Photosynthesis and the intracellular inorganic carbon pool in the bluegreen alga *Anabaena variabilis*: Response to external CO_2_ concentration. Planta.

[64] McKay RML, Gibbs SP, Espie GS (1993). Effect of dissolved inorganic carbon on the expression of carboxysomes, localization of Rubisco and the mode of inorganic carbon transport in cells of the cyanobacterium *Synechococcus* UTEX 625. Arch Microbiol.

[65] Price GD, Coleman JR, Badger MR (1992). Association of carbonic anhydrase activity with carboxysomes isolated from the cyanobacterium *Synechococcus* PCC7942. Plant Physiol.

[66] Whitney SM, Houtz RL, Alonso H (2011). Advancing our understanding and capacity to engineer nature’s CO_2_-sequestering enzyme. Rubisco Plant Physiol.

[67] Tcherkez GGB, Farquhar GD, Andrews TJ (2006). Despite slow catalysis and confused substrate specificity, all ribulose bisphosphate carboxylases may be nearly perfectly optimized. Proc Natl Acad Sci USA.

[68] Mueller-Cajar O, Whitney SM (2008). Directing the evolution of Rubisco and Rubisco activase: first impressions of a new tool for photosynthesis research. Photosynth Res.

[69] Zhang J, Liu G, Carvajal AI, Wilson RH, Cai Z, Li Y (2021). Discovery of a readily heterologously expressed Rubisco from the deep sea with potential for CO_2_ capture. Bioresour Bioprocess.

[70] Durão P, Aigner H, Nagy P, Mueller-Cajar O, Hartl FU, Hayer-Hartl M (2015). Opposing effects of folding and assembly chaperones on evolvability of Rubisco. Nat Chem Biol.

[71] Satagopan S, Huening KA, Tabita FR (2019). Selection of cyanobacterial (*Synechococcus* sp. strain PCC 6301) RubisCO variants with improved functional properties that confer enhanced CO_2_-dependent growth of *Rhodobacter capsulatus*, a photosynthetic bacterium. MBio..

[72] Atsumi S, Higashide W, Liao JC (2009). Direct photosynthetic recycling of carbon dioxide to isobutyraldehyde. Nat Biotechnol.

[73] Liang F, Lindblad P (2017). *Synechocystis* PCC 6803 overexpressing RuBisCO grow faster with increased photosynthesis. Metab Eng Commun.

[74] Iwaki T, Haranoh K, Inoue N, Kojima K, Satoh R, Nishino T (2006). Expression of foreign type I ribulose-1,5-bisphosphate carboxylase/ oxygenase (EC 4.1.1.39) stimulates photosynthesis in cyanobacterium *Synechococcus* PCC7942 cells. Photosynth Res..

[75] Takahashi H, Uchimiya H, Hihara Y (2008). Difference in metabolite levels between photoautotrophic and photomixotrophic cultures of *Synechocystis* sp PCC 6803 examined by capillary electrophoresis electrospray ionization mass spectrometry. J Exp Bot.

[76] Orthwein T, Scholl J, Spät P, Lucius S, Koch M, Macek B (2021). The novel PII-interactor PirC identifies phosphoglycerate mutase as key control point of carbon storage metabolism in cyanobacteria. Proc Natl Acad Sci.

[77] Janasch M, Asplund-Samuelsson J, Steuer R, Hudson EP (2019). Kinetic modeling of the Calvin cycle identifies flux control and stable metabolomes in *Synechocystis* carbon fixation. J Exp Bot.

[78] Liang F, Englund E, Lindberg P, Lindblad P (2018). Engineered cyanobacteria with enhanced growth show increased ethanol production and higher biofuel to biomass ratio. Metab Eng.

[79] Pettersen EF, Goddard TD, Huang CC, Couch GS, Greenblatt DM, Meng EC (2004). UCSF Chimera–a visualization system for exploratory research and analysis. J Comput Chem.

[80] Kiefer D, Merkel M, Lilge L, Henkel M, Hausmann R (2021). From acetate to bio-based products: Underexploited potential for industrial biotechnology. Trends Biotechnol.

[81] Qian X, Zhang Y, Lun DS, Dismukes GC (2018). Rerouting of metabolism into desired cellular products by nutrient stress: Fluxes reveal the selected pathways in cyanobacterial photosynthesis. ACS Synth Biol.

[82] Scholl J, Dengler L, Bader L, Forchhammer K (2020). Phosphoenolpyruvate carboxylase from the cyanobacterium *Synechocystis* sp. PCC 6803 is under global metabolic control by PII signaling. Mol Microbiol..

[83] Stal LJ, Moezelaar R (1997). Fermentation in cyanobacteria. FEMS Microbiol Rev.

[84] Gründel M, Scheunemann R, Lockau W, Zilliges Y (2012). Impaired glycogen synthesis causes metabolic overflow reactions and affects stress responses in the cyanobacterium *Synechocystis* sp. PCC 6803. Microbiology..

[85] Guerra LT, Xu Y, Bennette N, McNeely K, Bryant DA, Dismukes GC (2013). Natural osmolytes are much less effective substrates than glycogen for catabolic energy production in the marine cyanobacterium *Synechococcus* sp. strain PCC 7002. J Biotechnol..

[86] Monshupanee T, Incharoensakdi A (2014). Enhanced accumulation of glycogen, lipids and polyhydroxybutyrate under optimal nutrients and light intensities in the cyanobacterium *Synechocystis* sp. PCC 6803. J Appl Microbiol..

[87] Koch M, Berendzen KW, Forchhammer K (2020). On the role and production of polyhydroxybutyrate (PHB) in the cyanobacterium *Synechocystis* sp. PCC 6803. Life..

[88] Hauf W, Schlebusch M, Hüge J, Kopka J, Hagemann M, Forchhammer K (2013). Metabolic changes in *Synechocystis* PCC6803 upon nitrogen-starvation: Excess NADPH sustains polyhydroxybutyrate accumulation. Metabolites.

[89] Xu Y, Tiago Guerra L, Li Z, Ludwig M, Charles Dismukes G, Bryant DA (2013). Altered carbohydrate metabolism in glycogen synthase mutants of *Synechococcus* sp. strain PCC 7002: Cell factories for soluble sugars. Metab Eng..

[90] Damrow R, Maldener I, Zilliges Y (2016). The multiple functions of common microbial carbon polymers, glycogen and PHB, during stress responses in the non-diazotrophic cyanobacterium *Synechocystis* sp. PCC 6803. Front Microbiol..

[91] Luan G, Zhang S, Wang M, Lu X (2019). Progress and perspective on cyanobacterial glycogen metabolism engineering. Biotechnol Adv.

[92] Li X, Shen CR, Liao JC (2014). Isobutanol production as an alternative metabolic sink to rescue the growth deficiency of the glycogen mutant of *Synechococcus* elongatus PCC 7942. Photosynth Res.

[93] Velmurugan R, Incharoensakdi A (2018). Disruption of polyhydroxybutyrate synthesis redirects carbon flow towards glycogen synthesis in *Synechocystis* sp. PCC 6803 overexpressing glgC/glgA. Plant Cell Physiol..

[94] Wu GF, Wu QY, Shen ZY (2001). Accumulation of poly-β-hydroxybutyrate in cyanobacterium *Synechocystis* sp. PCC6803. Bioresour Technol..

[95] Osanai T, Shirai T, Iijima H, Nakaya Y, Okamoto M, Kondo A (2015). Genetic manipulation of a metabolic enzyme and a transcriptional regulator increasing succinate excretion from unicellular cyanobacterium. Front Microbiol.

[96] Yu J, Liberton M, Cliften PF, Head RD, Jacobs JM, Smith RD (2015). *Synechococcus elongatus* UTEX 2973, a fast growing cyanobacterial chassis for biosynthesis using light and CO_2_. Sci Rep.

[97] Włodarczyk A, Selão TT, Norling B, Nixon PJ (2020). Newly discovered *Synechococcus* sp. PCC 11901 is a robust cyanobacterial strain for high biomass production. Commun Biol..

[98] Matsusako T, Toya Y, Yoshikawa K, Shimizu H (2017). Identification of alcohol stress tolerance genes of *Synechocystis* sp. PCC 6803 using adaptive laboratory evolution. Biotechnol Biofuels.

[99] Zhang Y, Niu X, Shi M, Pei G, Zhang X, Chen L (2015). Identification of a transporter Slr0982 involved in ethanol tolerance in cyanobacterium *Synechocystis* sp. PCC 6803. Front Microbiol..

[100] Srivastava V, Srivastava V, Amanna R, Amanna R, Rowden SJL, Rowden SJL (2021). Adaptive laboratory evolution of the fast-growing cyanobacterium *Synechococcus elongatus* PCC 11801 for improved solvent tolerance. J Biosci Bioeng.

[101] Gonçalves AL, Rodrigues CM, Pires JCM, Simões M (2016). The effect of increasing CO_2_ concentrations on its capture, biomass production and wastewater bioremediation by microalgae and cyanobacteria. Algal Res.

[102] Huttenhuis P, Roeloffzen A, Versteeg G (2016). CO_2_ capture and re-use at a waste incinerator. Energy Procedia.

[103] Bonaventura D, Chacartegui R, Valverde JM, Becerra JA, Verda V (2017). Carbon capture and utilization for sodium bicarbonate production assisted by solar thermal power. Energy Convers Manage.

[104] Kumar K, Dasgupta CN, Nayak B, Lindblad P, Das D (2011). Development of suitable photobioreactors for CO_2_ sequestration addressing global warming using green algae and cyanobacteria. Bioresour Technol.

[105] Kamennaya NA, Ahn S, Park H, Bartal R, Sasaki KA, Holman H-Y (2015). Installing extra bicarbonate transporters in the cyanobacterium *Synechocystis* sp. PCC6803 enhances biomass production. Metab Eng..

[106] Gupta JK, Rai P, Jain KK, Srivastava S (2020). Overexpression of bicarbonate transporters in the marine cyanobacterium *Synechococcus* sp. PCC 7002 increases growth rate and glycogen accumulation. Biotechnol Biofuels..

[107] Stöckel J, Elvitigala TR, Liberton M, Pakrasi HB (2013). Carbon availability affects diurnally controlled processes and cell morphology of *Cyanothece* 51142. PLoS ONE..

[108] de Philippis R, Sili C, Vincenzini M (1996). Response of an exopolysaccharide-producing heterocystous cyanobacterium to changes in metabolic carbon flux. J Appl Phycol.

[109] Chou H-H, Su H-Y, Song X-D, Chow T-J, Chen C-Y, Chang J-S (2019). Isolation and characterization of *Chlorella* sp. mutants with enhanced thermo- and CO_2_ tolerances for CO_2_ sequestration and utilization of flue gases. Biotechnol Biofuels..

[110] Mackay MA, Norton RS, Borowitzka LJ (1984). Organic osmoregulatory solutes in cyanobacteria. Microbiology.

[111] Klähn S, Hagemann M (2011). Compatible solute biosynthesis in cyanobacteria. Environ Microbiol.

[112] Carrieri D, Momot D, Brasg IA, Ananyev G, Lenz O, Bryant DA (2010). Boosting autofermentation rates and product yields with sodium stress cycling: Application to production of renewable fuels by cyanobacteria. Appl Environ Microbiol.

[113] Koch M, Doello S, Gutekunst K, Forchhammer K (2019). PHB is produced from glycogen turn-over during nitrogen starvation in *Synechocystis* sp. PCC 6803. Int J Mol Sci..

[114] McNeely K, Xu Y, Bennette N, Bryant DA, Dismukes GC (2010). Redirecting reductant flux into hydrogen production via metabolic engineering of fermentative carbon metabolism in a cyanobacterium. Appl Environ Microbiol.

[115] Wu X-X, Li J-W, Xing S-F, Chen H-T, Song C, Wang S-G (2021). Establishment of a resource recycling strategy by optimizing isobutanol production in engineered cyanobacteria using high salinity stress. Biotechnol Biofuels.

[116] Schwarz R, Forchhammer K (2005). Acclimation of unicellular cyanobacteria to macronutrient deficiency: emergence of a complex network of cellular responses. Microbiology.

[117] Schlebusch M, Forchhammer K (2010). Requirement of the nitrogen starvation-induced protein Sll0783 for polyhydroxybutyrate accumulation in *Synechocystis* sp. strain PCC 6803. Appl Environ Microbiol..

[118] Carrieri D, Paddock T, Maness P-C, Seibert M, Yu J (2012). Photo-catalytic conversion of carbon dioxide to organic acids by a recombinant cyanobacterium incapable of glycogen storage. Energy Environ Sci.

[119] Russell JB (2007). The energy spilling reactions of bacteria and other organisms. J Mol Microbiol Biotechnol.

[120] Sun Y, Casella S, Fang Y, Huang F, Faulkner M, Barrett S (2016). Light modulates the biosynthesis and organization of cyanobacterial carbon fixation machinery through photosynthetic electron flow. Plant Physiol.

[121] Nogales J, Gudmundsson S, Knight EM, Palsson BO, Thiele I (2012). Detailing the optimality of photosynthesis in cyanobacteria through systems biology analysis. Proc Natl Acad Sci USA.

[122] Luimstra VM, Schuurmans JM, Verschoor AM, Hellingwerf KJ, Huisman J, Matthijs HCP (2018). Blue light reduces photosynthetic efficiency of cyanobacteria through an imbalance between photosystems I and II. Photosynth Res.

[123] Klepacz-Smółka A, Pietrzyk D, Szeląg R, Głuszcz P, Daroch M, Tang J (2020). Effect of light colour and photoperiod on biomass growth and phycocyanin production by *Synechococcus* PCC 6715. Bioresour Technol..

[124] Luimstra VM, Schuurmans JM, Hellingwerf KJ, Matthijs HCP, Huisman J (2020). Blue light induces major changes in the gene expression profile of the cyanobacterium *Synechocystis* sp. PCC 6803. Physiol Plant..

[125] Cano M, Holland SC, Artier J, Burnap RL, Ghirardi M, Morgan JA (2018). Glycogen synthesis and metabolite overflow contribute to energy balancing in cyanobacteria. Cell Rep.

[126] Sengupta T, Bhushan M, Wangikar PP. Metabolic modeling for multi-objective optimization of ethanol production in a Synechocystis mutant. Photosynth Res. 2013;118:155–65. 10.1007/s11120-013-9935-x 24190812

[127] Amer M, Wojcik EZ, Sun C, Hoeven R, Hughes JMX, Faulkner M (2020). Low carbon strategies for sustainable bio-alkane gas production and renewable energy. Energy Environ Sci.

[128] Chow T-J, Su H-Y, Tsai T-Y, Chou H-H, Lee T-M, Chang J-S (2015). Using recombinant cyanobacterium (*Synechococcus elongatus*) with increased carbohydrate productivity as feedstock for bioethanol production via separate hydrolysis and fermentation process. Bioresour Technol.

[129] Amer M, Hoeven R, Kelly P, Faulkner M, Smith MH, Toogood HS (2020). Renewable and tuneable bio-LPG blends derived from amino acids. Biotechnol Biofuels.

[130] Lopes TF, Cabanas C, Silva A, Fonseca D, Santos E, Guerra LT (2019). Process simulation and techno-economic assessment for direct production of advanced bioethanol using a genetically modified *Synechocystis* sp. Bioresour Technol.

[131] Madson PW, Lococo DB (2000). Recovery of volatile products from dilute high-fouling process streams. Appl Biochem Biotechnol.

[132] Yadav AN, Rastegari AA, Yadav N, Kour D (2020). Advances in plant microbiome and sustainable agriculture.

